# The Combined Contribution of Vascular Endothelial Cell Migration and Adhesion to Stent Re-endothelialization

**DOI:** 10.3389/fcell.2021.641382

**Published:** 2021-03-04

**Authors:** Xiaoli Wang, Fei Fang, Yinghao Ni, Hongchi Yu, Jia Ma, Li Deng, Chunli Li, Yang Shen, Xiaoheng Liu

**Affiliations:** West China School of Basic Medical Sciences and Forensic Medicine, Institute of Biomedical Engineering, Sichuan University, Chengdu, China

**Keywords:** in-stent restenosis, re-endothelialization, fluid shear stress, cell migration, cell adhesion

## Abstract

Coronary stent placement inevitably causes mechanical damage to the endothelium, leading to endothelial denudation and in-stent restenosis (ISR). Re-endothelialization depends mainly on the migration of vascular endothelial cells (VECs) adjacent to the damaged intima, as well as the mobilization and adhesion of circulating VECs. To evaluate the combined contribution of VEC migration and adhesion to re-endothelialization under flow and the influence of stent, *in vitro* models were constructed to simulate various endothelial denudation scales (2 mm/5 mm/10 mm) and stent deployment depths (flat/groove/bulge). Our results showed that (1) in 2 mm flat/groove/bulge models, both VEC migration and adhesion combined completed the percentage of endothelial recovery about 27, 16, and 12%, and migration accounted for about 21, 15, and 7%, respectively. It was suggested that the flat and groove models were in favor of VEC migration. (2) With the augmentation of the injury scales (5 and 10 mm), the contribution of circulating VEC adhesion on endothelial repair increased. Taken together, endothelial restoration mainly depended on the migration of adjacent VECs when the injury scale was 2 mm. The adhered cells contributed to re-endothelialization in an injury scale-dependent way. This study is helpful to provide new enlightenment for surface modification of cardiovascular implants.

## Introduction

Cardiovascular disease remains the leading cause of death globally (Kivimaki and Steptoe, 2018). Stent implantation reconstructs stenotic arteries by expanding the vascular wall and restoring blood flow perfusion. However, stent deployment inevitably causes endothelial denudation, which promotes in-stent restenosis (ISR) and late thrombosis (Krankenberg et al., 2015). Rapid re-endothelialization is an important therapeutic goal to avoid ISR and thrombosis (Liang et al., 2016; Bedair et al., 2017).

Endothelium restoration after stenting has been widely investigated (Tesfamariam, 2016). The repair of impaired vascular endothelium involves the migration of vascular endothelial cells (VECs) from adjacent uninjured sites and homing and adhesion of circulating VECs. However, the cell source of re-endothelialization is still being disputed (Van der Heiden et al., 2013). Since endothelial progenitor cells (EPCs) were found in peripheral blood, circulating EPCs were regarded as the primary cell source for reconstructing the damaged endothelium (Zhang et al., 2014). On the other hand, mounting evidence indicated that circulating EPCs (most likely monocytic) could not directly contribute to endothelial regeneration by forming part of the regenerating endothelium (Evans et al., 2020). As a marker of endothelial injury, circulating endothelial cells detached from impaired vessels, sloughed into the circulation, and contributed to vascular repair (Quilici et al., 2004; Blann et al., 2005). Hagensen et al. (2011) indicated that endothelial restoration mainly depended on the migration of VECs from the adjacent healthy endothelium. Douglas et al. (2013) found that both migration of VECs and adhesion of circulating cells participated in endothelial repair in stented arteries. Therefore, the contribution of adjacent VEC migration and circulating VEC adhesion to re-endothelialization needs further study.

There are several factors that can potentially influence endothelium recovery post stenting, including stent deployment depth, the scale of endothelial denudation, hemodynamic changes, and the structure and material properties of the stent (Kakinoki et al., 2018; Wang et al., 2018; Torii et al., 2020). Due to differences in individual vessel diameter and atherosclerotic plaque type, stent placement causes endothelium injury of various depths and scales (O’Brien et al., 2016). However, the effects of stent deployment depth and injury scales on adjacent VEC migration and circulating VEC adhesion remain poorly understood. Of note, if the diameter of the vessel is mismatched with stent expansion, stent strut is deeply embedded in a vessel well to form a “groove,” or part of the stent strut is exposed in the vessel lumen forming a “bulge” (insufficient expansion). In addition, the dilation of stent leads to almost complete endothelial loss of the stented segment, and only few endothelial cells remained at the edge of the stent (Douglas et al., 2013; Du et al., 2018). The average available stent length is longer than 10 mm, and the diameter of the stent ranges from 1 to 3 mm (Kalapatapu et al., 2007; Byrne et al., 2017). Accordingly, models with various scales were designed: (1) the thickness of a single stent strut (2 mm), (2) half of endothelial denudation at stent segment (5 mm), and (3) whole length of stented damaged to the vessel well (10 mm).

The presence of stent markedly alters vascular mechanics, especially blood flow patterns and fluid shear stress (FSS) (Gijsen et al., 2019; Torii et al., 2020). Under physiological conditions, endothelial cells are exposed to laminar FSS ranging from 10 to 20 dyn/cm^2^ (Kwak et al., 2014). Atherosclerotic plaque leads to arterial stenosis or occlusion, which increases FSS in the upstream and central plaque areas, while the downstream areas of plaque are exposed to low FSS (Michail et al., 2018). Implanted stent restores stenotic vessels and converts sharply elevated wall shear stress to relatively low level, but induces geometric changes of vascular wall and local turbulence (Van der Heiden et al., 2013). However, the influence of stent deployment depth and injury scales on local hemodynamics is still unclear.

In the present study, *in vitro* models were constructed to explore the effects of stent deployment depth and injury scales on local hemodynamics. Using these models, we further investigated the combined contribution of VEC migration and adhesion to re-endothelialization under flow. Our study will provide new inspiration for exploring the mechanism about stent re-endothelialization upon vascular biomechanical stimuli and modifying the surface of cardiovascular stent.

## Materials and Methods

### Construction of *in vitro* Injury Models After Stent Implantation

To explore the influence of stent on local hemodynamics and the combined contribution of VEC migration and adhesion to re-endothelialization, we constructed *in vitro* injury models to simulate various endothelial denudation scales (2 mm/5 mm/10 mm) and stent deployment depths (groove/flat/bulge). First, glass slides (7.5 × 2.5 × 1.0 cm) were used to build 2 mm/5 mm/10 mm embedment/protrusion, respectively (referred to as groove_2 mm_/groove_5 mm_/groove_10 mm_, bulge_2 mm_/bulge_5 mm_/Bulge_10 mm_, respectively).

### Characterization of Groove/Bulge Slide Models by SEM

The slides were dipped in a mixture of concentrated sulfuric acid and potassium dichromate overnight, cleaned to remove surface contaminants, dried, and then autoclaved. Sprayed with Au, the constructed groove, and bulge slide models were observed under a scanning electron microscope (SEM, Hitachi Company, Japan).

### Computational Fluid Dynamic Simulation

In this study, the straight left coronary artery was selected as the geometric structure of the vascular model based on the finite element method to calculate the hemodynamic and flow distribution of the coronary artery. ANSYS 16.0 (ANSYS, Inc., United States) was applied to divide finite element mesh and generate the finite element model. The input–output boundary of each finite element model was applied with the physiological boundary condition of the left coronary artery, and the control condition was set. The mean of the physiological parameters was applied in the numerical simulation. In detail, the vessel diameter was set as 4 mm, the inlet velocity was 0.5 m/s, the density of blood was 1,050 g/cm^3^, and the viscosity coefficient was 0.003 mPa s, respectively. Groove/bulge with a width of 2 mm/5 mm/10 mm and 0.085 mm/0.17 mm in depth was set in the vessels to simulate the different injury scales and stent deployment depths. Finally, the finite element method was used to solve the divided finite element model. After the solution was completed, the hemodynamic and flow distribution in different injury models after stent implantation were numerically analyzed.

### Cell Culture

Human umbilical vein endothelial cells (HUVECs) were chosen in the present study, which were purchased from Jiangsu Blood Research Institute. HUVECs were maintained in RPMI-1640 complete growth medium (Invitrogen Company, United States) with 10% fetal bovine serum (FBS, Gibco BRL, United States), 2 mM L-glutamine, 100 U/ml penicillin, 20 mmol/L HEPES (Sigma, United States), 2% NaHCO_3_, and 50 mg/ml streptomycin (Beyotime Institute of Biotechnology).

### Lentivirus-GFP/Lentivirus-mCherry Transfection of HUVECs

Lentivirus-GFP/Lentivirus-mCherry (Heyuan Biotechnology Co., Ltd., China) was used to transfect HUVECs with green fluorescent (GFP) and red fluorescent mCherry protein markers, respectively. HUVECs were grown in 24-well plates at a density of 7 × 10^4^ cells/well, then added with 500 μl RPMI-1640 complete growth medium and cultured at 37°C, in 5% CO_2_ incubator (Heraeus Company, Germany) for 24 h. Next, the culture medium was replaced with DMEM high-glucose complete medium (Gibco BRL, United States) with 5 μg/ml polybrene (Heyuan Biotechnology Co., Ltd., China) and preconfigured virus solution with MOI = 40. Polybrene is a cationic polymer, which can neutralize the electric charge to promote binding between the lentivirus and cell membrane. After 72 h of infection, the infection efficiency was evaluated by a fluorescence microscope (Olympus, Japan); 0.5 μg/ml purinomycin was selected to maintain HUVECs labeled with green fluorescent protein (GFP-HUVECs) or red fluorescent protein (mCherry-HUVECs). In the following experiments, GFP-HUVECs were used to characterize the migration of adjacent VECs, and mCherry-HUVECs to indicate the adhesion of circulating VECs.

### Cell Migration and Adhesion Assays

**FIGURE 1:**
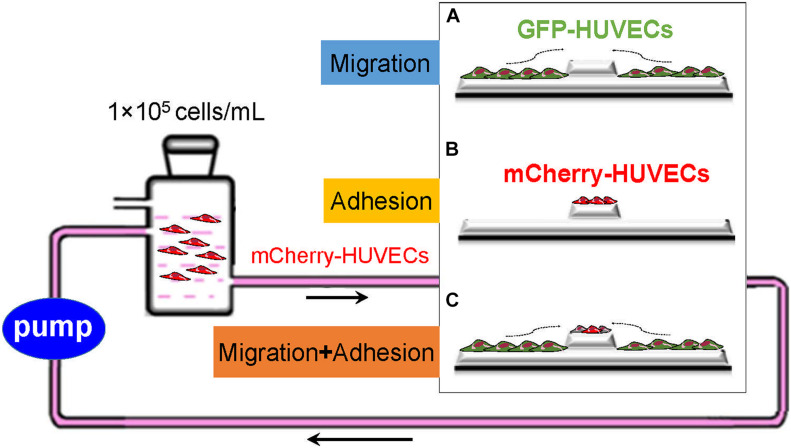
Schematic of exploring the cell sources of re-endothelialization and their contribution. GFP-HUVECs (green) and mCherry-HUVECs (red) were used to characterize adjacent VEC migration and adhesion of circulating VECs: **(A)** The migration of adjacent VECs contributed to re-endothelialization. **(B)** Circulatory VECs adhered to complete endothelium restoration. **(C)** The combined effect of adjacent VEC migration and circulatory VEC adhesion on stent re-endothelialization.

As shown in Figure 1A, the scratch-wound assay was used to measure the migration ability of HUVECs. (GFP-)HUVECs were cultured to approximately 90% confluence, and then a cell-free area was constructed at the embedment/protrusion part of groove_2 mm_/bulge_2 mm_ models and 2 mm scratches on flat slides (flat_2 mm_). Static culture or slides were placed into a parallel plate flow chamber to load 15.27 dyn/cm^2^ FSS for 24 h. The migrated distance/area of the cell was determined and calculated. As shown in Figure 1B, the adhesion assay was used to evaluate the adhesion ability of circulated HUVECs under static condition and FSS stimulation. The blank groove/bulge models were placed into static or circulated mCherry-HUVEC suspension (1 × 10^5^ cells/ml) for 24 h. The number/area of red fluorescent spots in each field of vision was analyzed. For the combined effect of cell migration and adhesion, the cell-free area on the embedment/protrusion part of the groove/bulge models after 90% confluence of (GFP-)HUVECs was constructed, and the mCherry-HUVEC suspension (1 × 10^5^ cells/ml) under static or dynamic condition for 24 h was introduced (as shown in Figure 1C). The cell migration distance/area and the adhesion number/area were quantitatively analyzed through ImageJ 1.44p software (National Institutes of Health, United States).

### F-Actin Staining

HUVECs on the groove_2 mm_ model were loaded with 15.27 dyn/cm^2^ FSS for 24 h and fixed with 4% paraformaldehyde (Biosharp Company, China) for 10 min at 37°C and then with 0.5% Triton X-100 for 5 min and 1% BSA block for 30 min. Then, the TRITC-labeled antibody F-actin (Solarbio Science & Technology Co., Ltd., China) with 1:200 dilution was added and co-incubated for 20 min at 37°C, followed by 1:800 diluted DAPI (4′,6′-diamidino-2-phenylindole) staining for 10 min at 37°C and washed with phosphate buffered saline (PBS). The samples sealed with glycerol were observed by laser scanning confocal microscopy (Leica TCS SP5, Germany).

### Cell Morphology by SEM

HUVECs were seeded onto 2 mm flat/groove/bulge models and cultured to approximately 90% confluence. Cells at the embedment/protrusion part of the groove/bulge models were removed. Then, cells were loaded with 15.27 dyn/cm^2^ laminar FSS for 24 h, followed by 0.1 M, pH 7.35, sodium arsenate solution (Budweiser Biotechnology Co., Ltd., China) containing 2% glutaraldehyde (Amerso Company, China) and 2% paraformaldehyde (Biosharp Company, China) fixation for 15 min, and washed for 5 min with PBS and distilled deionized water (ddH_2_O), respectively. The samples were dehydrated by 30, 50, 70, 90, and 100% ethanol solution and placed in a freeze dryer (Yaxing Yike Technology Co., Ltd., China). SEM (Hitachi Company, Japan) was used to observe the morphology of HUVECs.

### Statistical Analysis

All statistics were analyzed using statistical software SPSS 11.5 (SPSS, Inc., Chicago, Illinois). Data obtained from different treatment groups were statistically compared and reported as mean ± SD. To reveal differences among the groups, one-way ANOVA followed by Tukey’s test was used. Differences were considered significant at *P* < 0.05.

## Results

### Establishment of *in vitro* Vascular Injury Models After Stent Placement

**FIGURE 2:**
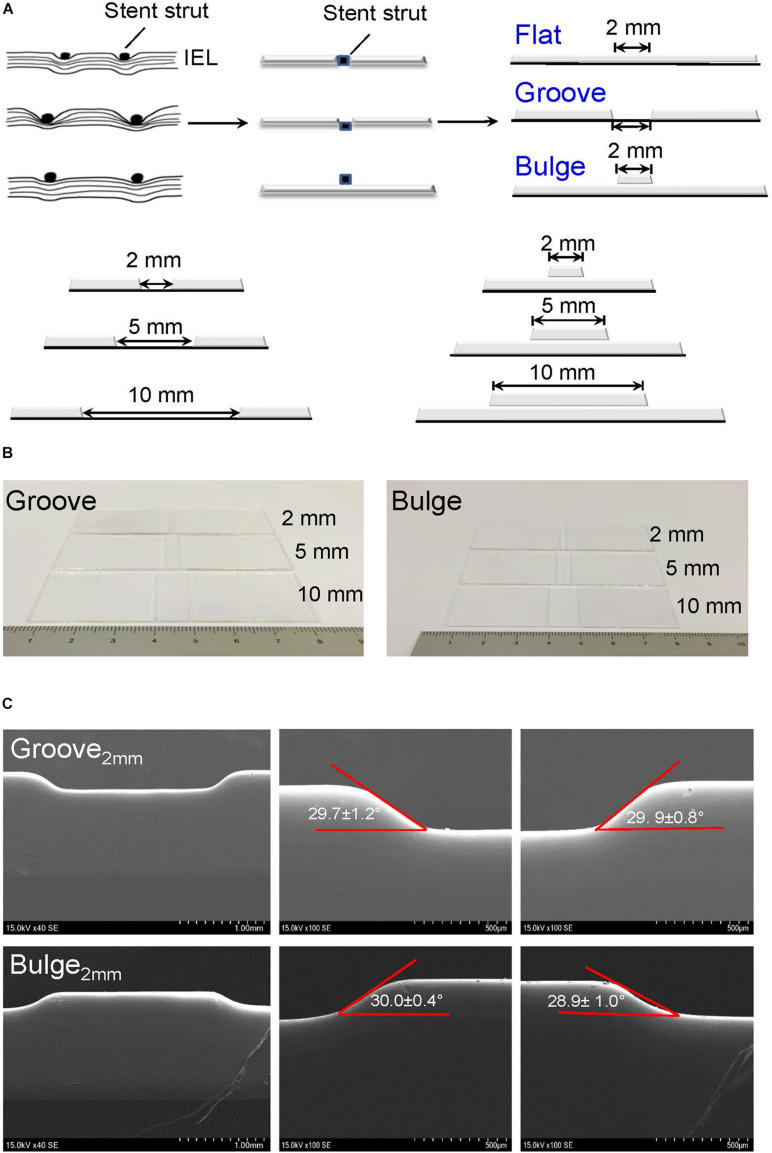
Construction of glass slide models to simulate vascular injury after stent implantation. **(A)** Design of vascular injury scales with 2 mm (the thickness of a single stent strut), 5 mm (half of endothelial denudation at stent segment), and 10 mm width (whole length of stent). The stent is embedded in the vessel wall with a horizontal line (flat model) or deeply embedded into the vessel wall (groove model); the stent is protruded from the vessel wall (bulge model). The black dots and squares represent stent struts embedded in the vessel wall. The curved lines indicate internal elastic lamina (IEL). **(B)** Typical digital images of groove/bulge models with widths of 2 mm/5 mm/10 mm. **(C)** Observation of edge angles at groove/bulge models with the width of 2 mm by SEM. Scale bars = 1 mm and 500 μm.

To simulate sufficient/insufficient expansion of stent *in vitro*, glass slides with embedment (thereafter referred to as groove) and protrusion (thereafter referred to as bulge) in injured scales of 2 mm/5 mm/10 mm were designed and constructed (Figures 2A,B). We verified that the depth of the groove model (2 mm) was 197.0 ± 6.8 μm. The left-edge angle (29.7 ± 1.2°) and the right-edge angle (29.9 ± 0.8°) were measured by SEM (Figure 2C, up panel). In the bulge model, the height of protrusion was 185.9 ± 2.8 μm, and the left- and right-edge angles were 30.0 ± 0.4° and 28.9 ± 1.0°, respectively (Figure 2C, down panel).

### Numerical Simulation of Hemodynamics and Flow Distribution

ANSYS 16.0 was used to simulate and calculate the effects of different stent deployment depth and injury scales on hemodynamics and flow distribution. The straight left main coronary artery was selected as the geometric structure of the vascular model. According to the physiology parameter of the left coronary artery, the vessel diameter was set as 4 mm, the inlet velocity was 0.5 m/s, the blood density was 1,050 g/cm^3^, and the viscosity coefficient was 0.003 mPa s, respectively.

**FIGURE 3:**
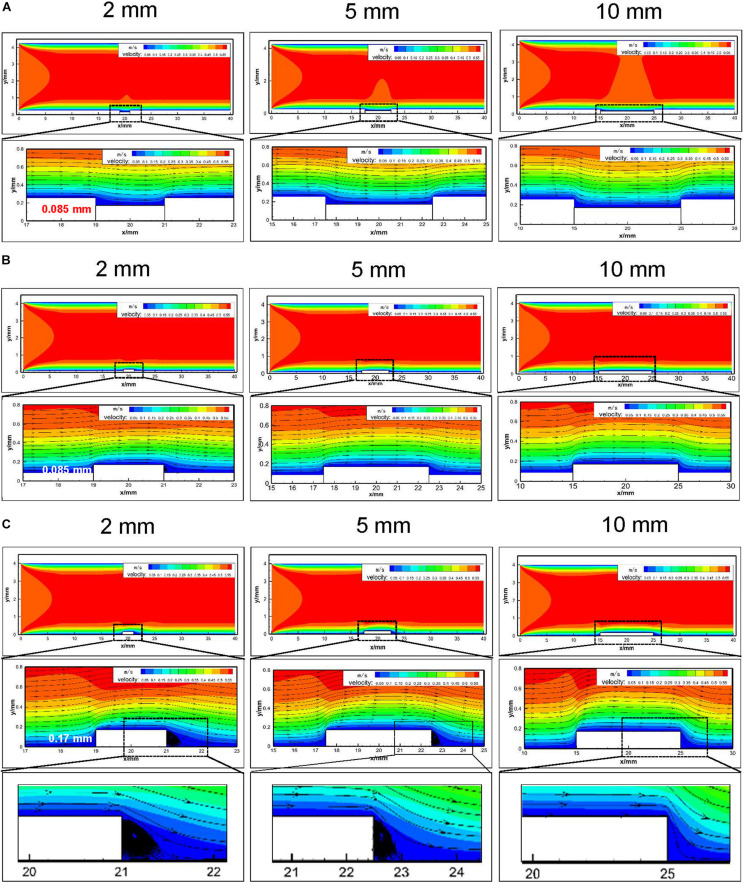
Numerical analysis of hemodynamics and flow distribution in embedment models with different injury scales (2 mm/5 mm/10 mm). **(A)** Embedment with 0.085 mm in depth and the hemodynamics and flow distribution of the injured blood vessels; **(B)** protrusion with 0.085 mm in height in the vessel lumen and the hemodynamics and flow distribution of the injured vessel segment; **(C)** protrusion with 0.17 mm in height in the vessel lumen and the hemodynamics and flow distribution of the injured blood vessels. The enlarged black area indicated that downstream of the 2 and 5 mm bulge model caused fluid separation area and flow turbulence.

It could be found that the overall flow distribution was not affected by stent placement (Figure 3). In the groove models simulating sufficient stent dilation, the scale of vascular damage was set as 2, 5, and 10 mm with 0.085 mm in depth (Figure 3A). There was no flow disturbance in the upstream and downstream of the damaged vessel segment. In the models simulating stent expansion with proper pressure, the height of the stent protrusion was 0.085 mm. An obvious disturbance appeared in the downstream of 2 mm width; when the damage scale was up to 5 and 10 mm, the disturbance flow disappeared (Figure 3B). In the bulge models simulating insufficient stent expansion, the scale of vascular damage was set as 2, 5, and 10 mm with 0.17 mm in height. The downstream of 2 and 5 mm width caused fluid separation area and obvious flow turbulence. When the injury scale increased to 10 mm, the flow disturbance disappeared (Figure 3C). These results implied that stent-induced injury had no obvious influence on the upstream flow distribution but had a significant influence on the downstream flow, depending on the injury scales and height of the stent.

### Cell Sources and the Respective Contribution to Re-endothelialization Under Static Conditions

**FIGURE 4:**
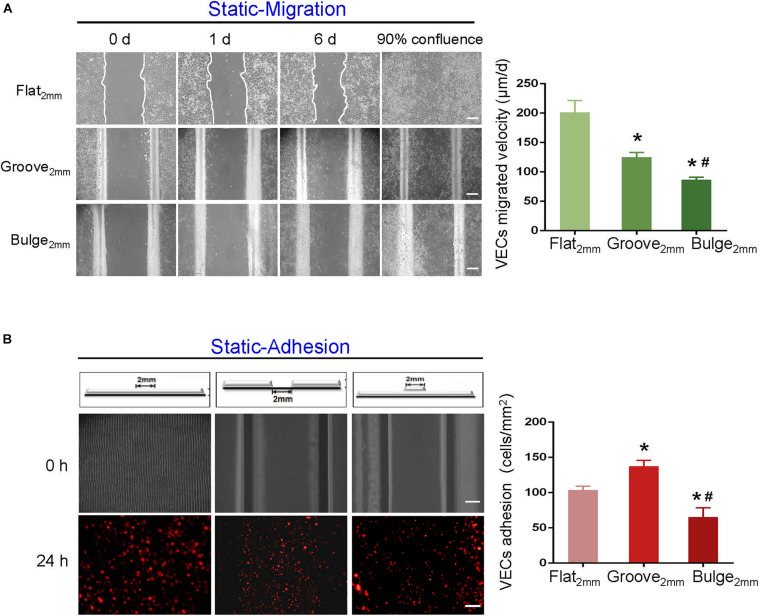
Effects of groove/bulge models on VEC migration or adhesion under static condition. Under static condition, **(A)** groove_2 mm_/bulge_2 mm_ models were cultured until 90% confluence, and then cells were removed on the embedment/protrusion part, investigating the effects of stent embedment/protrusion on VEC migration and its statistical results. **(B)** Blank groove_2 mm_/bulge_2 mm_ models were placed into 1 × 10^5^ cells/ml mCherry-HUVEC suspension for 24 h, determining the effects of stent embedment/protrusion on VEC adhesion and its statistical results. *P < 0.05 vs. flat_2 mm_; #*P* < 0.05 vs. groove_2 mm_, the difference was statistically significant (*n* = 3). Scale bar = 500 μm.

Using scratch-wound assays, we investigated the effects of groove/bulge models with 2 mm width (referred to as groove_2 mm_/bulge_2 mm_) on the adjacent VEC migration and re-endothelialization under static conditions. The 2 mm scratch on glass slides (flat_2 mm_) was constructed as the control group. Under static condition, 90% of wound was healed in the flat_2 mm_/groove_2 mm_/bulge_2 mm_ models at 8, 16, and 24 days, respectively (Figure 4A, left panel), suggesting that the migration velocity of VECs in the flat_2 mm_ model was faster than that in the groove_2 mm_ model and the bulge_2 mm_ model (Figure 4A, right panel). The number of adhesive VECs per unit area in flat_2 mm_/groove_2 mm_/bulge_2 mm_ was 126 ± 5, 142 ± 7, and 68 ± 11 after 24 h, which was groove_2 mm_ > flat_2 mm_ > bulge_2 mm_ (Figure 4B).

**FIGURE 5:**
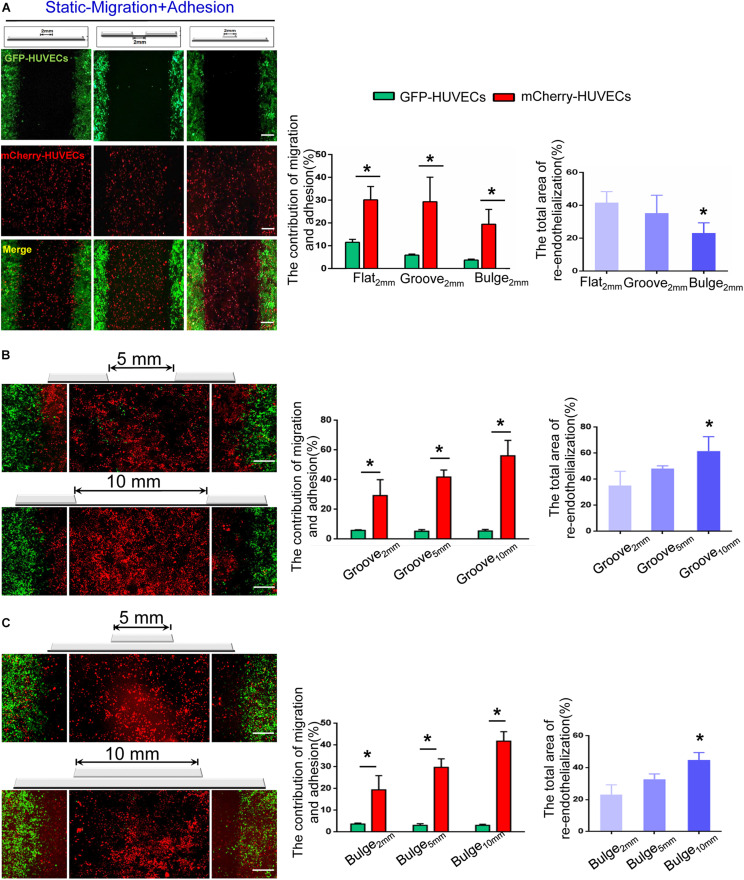
The combined effect of VEC migration and adhesion on re-endothelialization under static condition. **(A)** In the flat_2 mm_/groove_2 mm_/bulge_2 mm_ models, the endothelial cell source of endothelium recovery and their respective contributions were analyzed. *P < 0.05 vs. flat_2 mm,_ the difference was statistically significant (*n* = 3). **(B,C)** With the augmentation of the injury scales to 5 and 10 mm in the groove/bulge models, the endothelial cell sources of re-endothelialization were analyzed. *P < 0.05 vs. groove_2 mm_ or bulge_2 mm_, the difference was statistically significant (*n* = 3). Scale bar = 500 μm.

Additionally, we further examined the combined effect of VEC migration and adhesion and analyzed their respective contribution to re-endothelialization. The results indicated that the adhesion of mCherry-HUVECs was the main cell source for damaged endothelium repair under static condition. In the flat_2 mm_/groove_2 mm_/bulge_2 mm_ model, endothelium recovery in flat_2 mm_ was 41%, adjacent VEC migration accounted for 11%, and the percentage of adherent VECs was 30%. In the groove_2 mm_ model, approximately 59% endothelium restoration was completed, the migrated cells accounted for 6%, while the adherent cells contributed 29%. In the bulge_2 mm_ model, the percentage of re-endothelialization was 23%, the migration was 4%, and the adhesion was 19% (Figure 5A).

With increased injury scales (5 and 10 mm), more adherent circulating VECs contributed to endothelial repair. In the groove model with widths of 5 and 10 mm, endothelium recovery was up to approximately 48 and 61%, and adhesion was about 43 and 56%, respectively (Figure 5B). In the bulge_5 mm_/bulge_10 mm_ models, the percentage of re-endothelialization was 33 and 45%, and adhesion accounted for 30 and 42%, respectively (Figure 5C). The results demonstrated that circulating VECs contributed to re-endothelialization in an endothelial denudation scale-dependent way under the static condition.

### Effects of Vascular Injury Model on VEC Morphology Under Flow

**FIGURE 6:**
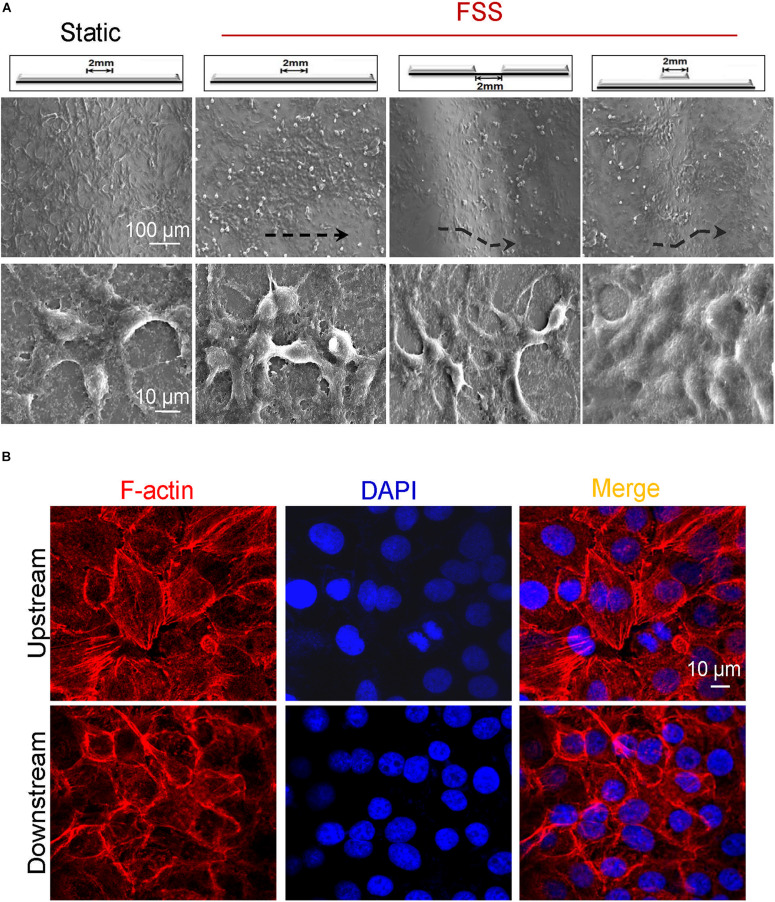
Morphology of endothelial cells in vascular injury models under flow. **(A)** Exposed to FSS, endothelial cells spread out with obvious parapodium in the groove models in favor of directed cell locomotion, while they accumulated in clusters on the edge of the bulge models. Scale bars = 100 and 10 μm. The black dashed lines indicated the flow direction. **(B)** Exposed to FSS, the distribution of F-actin arrays in VECs upstream and downstream of the groove_2 mm_ model. Scale bar = 10 μm.

The influence of flat_2 mm_/groove_2 mm_/bulge_2 mm_ on VEC morphology under flow was observed by SEM. As shown in Figure 6A, compared with static control, FSS promoted endothelial cells to arrange along the shear direction. In response to FSS exposure, endothelial cells spread out with obvious parapodium in groove_2 mm_ models in favor of directed cell locomotion, while VECs accumulated in clusters on the edge of bulge_2 mm_ models. Additionally, in the groove_2 mm_ model, the distribution of F-actin in VECs exposed to FSS was examined. The confocal images indicated that VECs upstream showed longer filopodia and more bundles of filaments at the leading edge of the cells. By contrast, VECs downstream displayed disordered actin stress fibers (Figure 6B).

### Cell Sources of Re-endothelialization and Their Contribution Under Flow

To explore the combined contribution of VEC migration and adhesion to re-endothelialization under flow, the labeled GFP-HUVECs and circulating mCherry-HUVECs were placed in a flow chamber together. The GFP-HUVECs were firstly cultured onto flat_2 mm_/groove_2 mm_/bulge_2 mm_ models until confluence, and subsequently, the cell-free area of the embedment/protrusion part was constructed. These models were then placed in the parallel plate flow chamber with circulated mCherry-HUVEC suspension (1 × 10^5^ cells/ml) in the perfusion system for 24 h.

**FIGURE 7:**
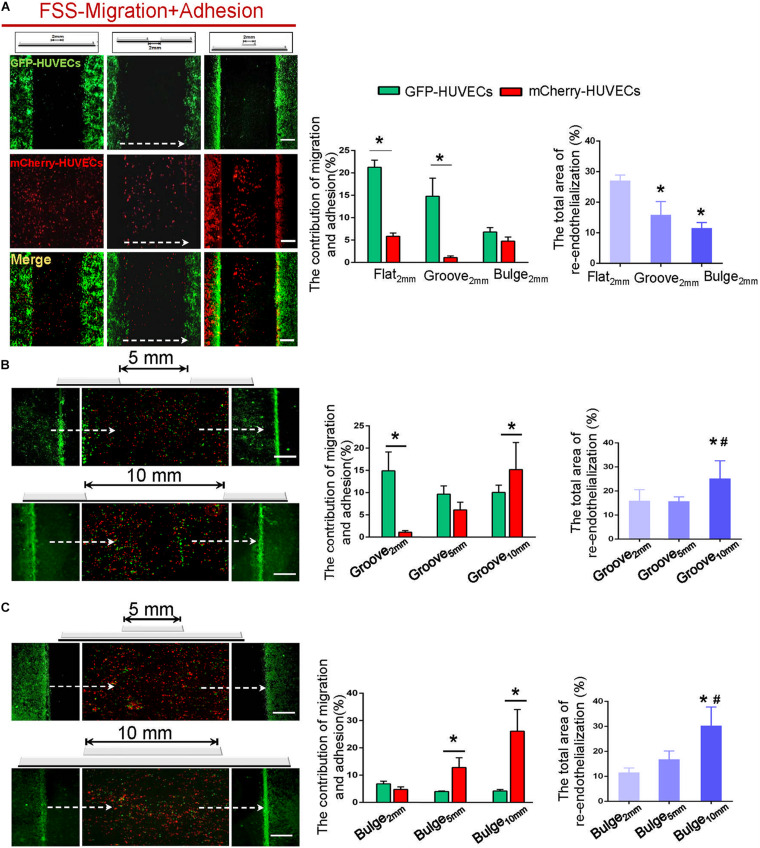
The combined contribution of VEC migration and adhesion to re-endothelialization under flow. **(A)** In the flat_2 mm_/groove_2 mm_/bulge_2 mm_ models, the endothelial cell source of endothelial restoration and their respective contribution under flow were analyzed. **(B,C)** With the augmentation of the injury scales to 5 and 10 mm in the groove/bulge models, the endothelial cell source of re-endothelialization under flow was analyzed. The white dashed lines indicated the flow direction. *P < 0.05 vs. flat_2 mm_ or groove_2 mm_ or bulge_2 mm_; ^#^*P* < 0.05 vs. groove_5 mm_ or bulge_5 mm_, the difference was statistically significant (*n* = 3). Scale bar = 500 μm.

It could be found that approximately 27% (flat_2 mm_), 16% (groove_2 mm_), and 12% (bulge_2 mm_) of re-endothelialization were completed. The migrated VECs (GFP-HUVECs) accounted for 21% (flat_2 mm_), 15% (groove_2 mm_), and 7% and the adhered VECs (mCherry-HUVECs) were 6% (flat_2 mm_), 1% (groove_2 mm_), and 5% (bulge_2 mm_), respectively (Figure 7A). These results indicated that the flat and groove models promoted VEC migration under flow, while the bulge model facilitated cell adhesion.

With increased injury scales (5 and 10 mm), more adherent circulating VECs could be found, in turn largely contributing to endothelial repair in an endothelial denudation scale-dependent way. In the groove model with widths of 5 and 10 mm, impaired endothelium recovered approximately 16 and 25%, and the adhesion was about 6 and 15%, respectively (Figure 7B). In the bulge_5 mm_/bulge_10 mm_ models, the percentage of re-endothelialization was 17 and 30%, and the adhesion was 13 and 26% (Figure 7C). The results indicated that adjacent VEC migration was the main contributor of endothelial restoration under flow in models with 2 mm injury width. The increased injury scale promoted the adherent cells to contribute to endothelial regeneration.

## Discussion

Interventions including balloon angioplasty and stent implantation inevitably cause mechanical damage to the endothelium, leading to endothelial denudation and subsequent ISR and late thrombosis. The pathophysiological mechanism of ISR has not been fully elucidated, but it is considered to include inflammation, proliferation, and matrix remodeling. Rapid restoration of functional vascular endothelium is an important therapeutic goal to avoid ISR, late thrombosis, and other postoperative complications (Bedair et al., 2017; Chang et al., 2018).

Factors including stent deployment depth, the scale of endothelial denudation, hemodynamic changes, and the structure and material properties of the stent potentially regulate re-endothelialization in stented vessels (Conway and Schwartz, 2015; Kang et al., 2015; Liang et al., 2016; Ostrowski et al., 2016). Due to the lack of effective *in vivo* and *in vitro* models, the cell sources of endothelial repair after stent implantation have not been fully understood. In this study, we developed an *in vitro* vascular injury model to mimic various endothelial denudation scales (2 mm/5 mm/10 mm) and stent deployment depths (groove/flat/bulge) (Figures 2A,B). The models of vascular injury *in vitro* were validated by SEM, and our results revealed that in the groove model, the deployment depth was 197.0 ± 6.8 μm, and the left- and right-edge angles were 29.7 ± 1.2° and 29.9 ± 0.8°, respectively. In the bulge model, the protrusion height was 185.9 ± 2.8 μm, and the left- and right-edge angles were 30.0 ± 0.4° and 28.9 ± 1.0°, respectively (Figure 2C).

The presence of stent inside the blood vessel causes changes in the local flow environment (Ng et al., 2017; Tenekecioglu et al., 2017). To evaluate the influence of different deployment depths and endothelial denudation scales on flow distribution, computational fluid dynamics simulation analysis was carried out. Our results suggested that groove/bulge models did not alter the hemodynamics in the upstream of stent segment, while they generated flow separation in the downstream and led to turbulence, depending on the injury scales and stent deployment depth (Figure 3). Consistent with our results, Jiang et al. (2019) revealed that stent deployment led to local flow turbulence and fluid separation area at the distal end of stent struts, which was characterized by flow recirculation, low shear rate, and long particle residence time. Of note, it is hard to simulate all physiological parameters at the same time in numerical simulation, and some conditions may be simplified. In the present study, we performed a two-dimensional simulation, which neglected some details that could be observed in three-dimensional simulation, such as blood vessel wall pressure, wall shear stress (WSS), and other hemodynamic parameters. Therefore, the changes of WSS in the groove and bulge models were not calculated and analyzed. By placing undersized stent at the lesion site or insufficient expansion, it was found that upstream of the stent induced high shear stress while the downstream produced low shear stress (Rikhtegar et al., 2014). Wang et al. (2018) indicated that low WSS was usually observed at the distal of the stent struts as compared with the proximal end of the struts. Using an *in vitro* flow chamber that contained ridges, Hsiao et al. (2016) indicated that flow rate of 21.6 ml/min medium would generate flow velocities that were the highest through the center (0.25 m/s). Additionally, the ridges created local flow disturbances including a region of very high unidirectional WSS (>100 dyn/cm^2^) at the top of the ridge and recirculating bidirectional flow with low-average WSS downstream from the ridge.

Increasing evidence pointed out that the degree of stent-induced arterial injury was correlated to re-endothelialization rate (Gunn et al., 2002; Gao et al., 2015). However, the effect of stent deployment depth and injury scales on endothelial restoration remains unclear. Based on the constructed flat_2 mm_/groove_2 mm_/bulge_2 mm_ models, we investigated the effects of stent deployment depth on adjacent VEC migration, circulating VEC adhesion, and their combined effect on re-endothelialization under static condition. As shown in Figures 4, 5, our results indicated that the groove model was in favor of VEC migration and adhesion, and re-endothelialization was mainly derived from the adhesion of VECs under static condition. Consistently, Palmaz et al. (1999) found that grooved surfaces significantly increased the migration rate of endothelial cells. A novel micro/nanopatterned scaffold surface with a typical geometry of groove, ranging from 0.5 to 50 μm, was developed to evaluate cell reactions. Their results revealed that groove could selectively promote endothelial repair and inhibit the proliferation of SMCs in a width-dependent manner (Ding et al., 2014). Additionally, with stent deployment depths of 90, 110, and 130 μm, Tahir et al. (2013) showed that the deeper penetration of stent struts resulted in a late endothelial recovery and higher neointimal growth. Further, they qualitatively compared the restoration of damaged intima at a deployment depth of 110 μm, which found approximately 59% endothelium presented from the third day after stenting, and endothelium recovery was 100% after 15 days.

Stent application results in geometric changes of vascular wall and disturbed flow, which regulate the physiology of VECs. VECs showed resistance to vascular injury under high laminar FSS, while they expressed proinflammatory and prothrombotic genes and adopt an athero-prone phenotype when exposed to low and oscillating flow (Souilhol et al., 2020). Here, we explored the effects of the vascular injury model on VEC morphology under flow. It was found that VECs spread out with obvious parapodium in groove models in favor of directed cell locomotion, while they accumulated in clusters on the edge of bulge models (Figure 6A). Additionally, endothelial cells exposed to FSS exhibited stress filament formation upstream of groove_2 mm_, while they displayed disordered actin stress fibers downstream of groove_2 mm_ (Figure 6B). The difference in the morphologies of VECs upstream and downstream may be caused by the changes of flow distribution and wall shear stress. Yoshino et al. (2017) showed that a higher WSS flow needed a higher WSS gradient to be able to affect the cell’s morphological change. Instead, on lower uniform WSS without WSS gradient flow, EC elongation and reorientation to the flow direction occurred.

Re-endothelialization is a complex mechanobiological process, which is modulated by the proliferation and migration of resident endothelial cells from uninjured intima (Evans et al., 2020; Li et al., 2021) and by the adhesion of circulating endothelial cells (Tesfamariam, 2016; Hu et al., 2019). Increased circulating endothelial cells in the peripheral blood have been reported in various pathologic conditions involving severe endothelial perturbation, including inflammatory disease, acute myocardial infarction, unstable angina, and critical limb ischemia (Makin et al., 2004; Lee et al., 2005). Using different pathological animal models and mechanical damage models in various studies, the cell source of endothelial repair after vascular injury has been controversial. By grafting the common carotid artery of transgenic mice with fluorescent endothelium (Tie2-GFP) into wild-type mice, Hagensen et al. (2011) illustrated that the migration but not the adhesion of endothelial cells contributed to the regeneration of the endothelium. However, using mice that received either aortic or bone marrow grafts from transgenic mice with Tie2-LacZ-labeled endothelial cell, another study revealed that both local VEC migration and circulating cell adhesion participated in endothelium restoration, although the respective contribution varied between animals (Douglas et al., 2013).

Combining stent-induced vascular injury with mechanical factors, we further explored the combined effect of VEC migration and adhesion on re-endothelialization and their contribution. It could be seen from Figure 7 that endothelial repair mainly depended on the migration of adjacent VECs at an injury scale of 2 mm, while the quantity of circulating VEC adhesion increased and largely contributed to endothelial repair with the increase of the injury scale, showing an injury scale dependence. A possible explanation for this phenomenon is the different WSS in the groove and bulge models. Alterations in flow patterns and WSS induced by stent application have been correlated with VEC migration and adhesion (Putra et al., 2018; Wang et al., 2018). Consistently, using a ridged flow chamber, Hsiao et al. (2016) demonstrated that VECs migrated in the direction of flow upstream from the ridges but subsequently accumulated downstream from ridges at sites of bidirectional flow. Localized bidirectional flow in the downstream of the stent trapped migrating VECs, which involved reduced migratory polarity associated with altered actin dynamics. Ostrowski et al. (2014) observed that human microvascular ECs were stimulated to migrate toward the region of high WSS and against the flow direction under the influence of WSS gradient distribution created by the impinging flow. Additionally, employing a lab-on-a-chip system, Stamp et al. (2016) systematically investigated cell adhesion under static, dynamic, and physiologically relevant conditions. They found increased detachment with increasing surface roughness under dynamic conditions, which involved shear flow-induced activation of focal adhesions, leading to an enhancement of stress fibers.

In conclusion, we successfully constructed an *in vitro* injury model to simulate various endothelial denudation scales and stent deployment depths. Flow distribution analysis revealed that the injury models did not alter the hemodynamics in the upstream of stent segment but generated flow separation in the downstream and led to turbulence, depending on the injury scales and stent deployment depth. Furthermore, this study preliminarily clarified the endothelial cell sources of re-endothelialization mainly derived from the migration of adjacent VECs when the injury scale was 2 mm; with the increase of the injury scale, the contribution of VEC adhesion to endothelium restoration increased in an injury scale-dependent way. Our study will provide new enlightenment for researchers engaged in vascular biomechanics and surface modification of cardiovascular implants.

## Data Availability Statement

The original contributions presented in the study are included in the article/supplementary material, further inquiries can be directed to the corresponding author/s.

## Ethics Statement

This article does not contain any studies with human participants or animals performed by the any of the authors.

## Author Contributions

XW, YS, and XL were responsible for the conception and design, acquisition, analysis, and interpretation of data, and drafting of the manuscript. FF and YN were responsible for SEM and numerical simulation data acquisition. HY was responsible for statistics. JM, LD, and CL were responsible for cell migration and adhesion assay data acquisition. YS and XL were responsible for revising the manuscript critically for important intellectual content and for the final approval of the version to be published. All authors read and approved the final manuscript.

## Conflict of Interest

The authors declare that the research was conducted in the absence of any commercial or financial relationships that could be construed as a potential conflict of interest.

## References

[B1] Bedair T. M. ElNaggar M. A. Joung Y. K. Han D. K. (2017). Recent advances to accelerate re-endothelialization for vascular stents. *J. Tissue Eng.* 8 1–14. 10.1177/2041731417731546 28989698 PMC5624345

[B2] Blann A. D. Woywodt A. Bertolini F. Bull T. M. Buyon J. P. Clancy R. M. (2005). Circulating endothelial cells. *Biomarker Vasc. Dis. Thromb. Haemost* 93 228–235. 10.1160/TH04-09-0578 15711737

[B3] Byrne R. A. Stone G. W. Ormiston J. Kastrati A. J. T. L. (2017). Coronary balloon angioplasty, stents, and scaffolds. *Lancet* 390 781–792. 10.1016/S0140-6736(17)31927-X 28831994

[B4] Chang H. K. Kim P. H. Kim D. W. Cho H. M. Jeong M. J. Kim D. H. (2018). Coronary stents with inducible VEGF/HGF-secreting UCB-MSCs reduced restenosis and increased re-endothelialization in a swine model. *Exp. Mol. Med.* 50:114. 10.1038/s12276-018-0143-9 30174328 PMC6119684

[B5] Conway D. E. Schwartz M. A. (2015). Mechanotransduction of shear stress occurs through changes in VE-cadherin and PECAM-1 tension: implications for cell migration. *Cell Adhes. Migr.* 9 335–339. 10.4161/19336918.2014.968498 25482618 PMC4955370

[B6] Ding Y. Yang Z. Bi C. W. Yang M. Xu S. L. Lu X. (2014). Directing vascular cell selectivity and hemocompatibility on patterned platforms featuring variable topographic geometry and size. *ACS Appl. Mater. Interfaces* 6 12062–12070. 10.1021/am502692k 25039647

[B7] Douglas G. Van Kampen E. Hale A. B. McNeill E. Patel J. Crabtree M. J. (2013). Endothelial cell repopulation after stenting determines in-stent neointima formation: effects of bare-metal vs. drug-eluting stents and genetic endothelial cell modification. *Eur. Heart J.* 34 3378–3388. 10.1093/eurheartj/ehs240 23008511 PMC3827553

[B8] Du R. Wang Y. Huang Y. Zhao Y. Zhang D. Du D. (2018). Design and testing of hydrophobic core/hydrophilic shell nano/micro particles for drug-eluting stent coating. *NPG Asia Mater.* 10 642–658. 10.1038/s41427-018-0064-z

[B9] Evans C. E. Iruela-Arispe M. L. Zhao Y.-Y. (2020). Mechanisms of endothelial regeneration and vascular repair and their application to regenerative medicine. *Am. J. Pathol.* 191 52–65. 10.1016/j.ajpath.2020.10.001 33069720 PMC7560161

[B10] Gao M. Yao Q. Liu Y. Sun F. Ma Y. Sun G. (2015). Association between mobilization of circulating endothelial progenitor cells and time or degree of injury from angioplasty in patients with exertional angina: a prospective study. *Exp. Ther. Med.* 10 809–815. 10.3892/etm.2015.2571 26622398 PMC4509458

[B11] Gijsen F. Katagiri Y. Barlis P. Bourantas C. Collet C. Coskun U. (2019). Expert recommendations on the assessment of wall shear stress in human coronary arteries: existing methodologies, technical considerations, and clinical applications. *Eur. Heart J.* 40 3421–3433. 10.1093/eurheartj/ehz551 31566246 PMC6823616

[B12] Gunn J. Arnold N. Chan K. H. Shepherd L. Cumberland D. C. Crossman D. C. (2002). Coronary artery stretch versus deep injury in the development of in-stent neointima. *Heart* 88 401–405. 10.1136/heart.88.4.401 12231603 PMC1767367

[B13] Hagensen M. K. Raarup M. K. Mortensen M. B. Thim T. Nyengaard J. R. Falk E. (2011). Circulating endothelial progenitor cells do not contribute to regeneration of endothelium after murine arterial injury. *Cardiovasc. Res.* 93 223–231. 22012957 10.1093/cvr/cvr278

[B14] Hsiao S. T. Spencer T. Boldock L. Prosseda S. D. Xanthis I. Tovar-Lopez F. J. (2016). Endothelial repair in stented arteries is accelerated by inhibition of Rho-associated protein kinase. *Cardiovasc. Res.* 112 689–701. 10.1093/cvr/cvw210 27671802 PMC5157135

[B15] Hu Q. Ke X. Zhang T. Chen Y. Huang Q. Deng B. (2019). Hydrogen sulfide improves vascular repair by promoting endothelial nitric oxide synthase-dependent mobilization of endothelial progenitor cells. *J. Hypertens.* 37 972–984. 10.1097/HJH.0000000000001983 30489453

[B16] Jiang B. Thondapu V. Poon E. Barlis P. Ooi A. (2019). Numerical study of incomplete stent apposition caused by deploying undersized stent in arteries with elliptical cross-sections. *J. Biomech. Eng.* 141:054501. 10.1115/1.4042899 30778567

[B17] Kakinoki S. Takasaki K. Mahara A. Ehashi T. Hirano Y. Yamaoka T. (2018). Direct surface modification of metallic biomaterials via tyrosine oxidation aiming to accelerate the re-endothelialization of vascular stents. *J. Biomed. Mater. Res. A* 106 491–499. 10.1002/jbm.a.36258 28975703

[B18] Kalapatapu K. Dilmanian H. Aronow W. S. Mundia M. Pucillo A. L. Weiss M. B. (2007). The average stent length is longer and the average stent diameter is shorter in patients with drug-eluting stents vs bare-metal stents during percutaneous coronary intervention. *Am. J. Ther.* 14 277–279. 10.1097/MJT.0b013e3180653377 17515704

[B19] Kang T.-Y. Lee J. H. Kim B. J. Kang J.-A. Hong J. M. Kim B. S. (2015). In vivo endothelization of tubular vascular grafts through in situ recruitment of endothelial and endothelial progenitor cells by RGD-fused mussel adhesive proteins. *Biofabrication* 7:015007. 10.1088/1758-5090/7/1/015007 25599716

[B20] Kivimaki M. Steptoe A. (2018). Effects of stress on the development and progression of cardiovascular disease. *Nat. Rev. Cardiol.* 15 215–229. 10.1038/nrcardio.2017.189 29213140

[B21] Krankenberg H. Tübler T. Ingwersen M. Schlüter M. Scheinert D. Blessing E. (2015). Drug-coated balloon versus standard balloon for superficial femoral artery in-stent restenosis: the randomized femoral artery in-stent restenosis (FAIR) trial. *Circulation* 132 2230–2236. 10.1161/CIRCULATIONAHA.115.017364 26446728

[B22] Kwak B. R. Magnus B. C. Marie-Luce B. P. Giuseppina C. Daemen M. J. A. P. Davies P. F. (2014). Biomechanical factors in atherosclerosis: mechanisms and clinical implications. *Eur. Heart J.* 35 3013–3020. 10.1093/eurheartj/ehu353 25230814 PMC4810806

[B23] Lee K. W. Lip G. Y. Tayebjee M. Foster W. Blann A. D. (2005). Circulating endothelial cells, von Willebrand factor, interleukin-6, and prognosis in patients with acute coronary syndromes. *Blood* 105 526–532. 10.1182/blood-2004-03-1106 15374879

[B24] Li J. Chen Y. Gao J. Chen Y. Zhou C. Lin X. (2021). Eva1a ameliorates atherosclerosis by promoting re-endothelialization of injured arteries via Rac1/Cdc42/Arpc1b. *Cardiovasc. Res.* 117 450–461. 10.1093/cvr/cvaa011 31977009

[B25] Liang C. Hu Y. Wang H. Xia D. Li Q. Zhang J. (2016). Biomimetic cardiovascular stents for in vivo re-endothelialization. *Biomaterials* 103 170–182. 10.1016/j.biomaterials.2016.06.042 27380443

[B26] Makin A. J. Blann A. D. Chung N. A. Silverman S. H. Lip G. Y. (2004). Assessment of endothelial damage in atherosclerotic vascular disease by quantification of circulating endothelial cells. Relationship with von Willebrand factor and tissue factor. *Eur. Heart J.* 25 371–376. 10.1016/j.ehj.2003.04.001 15033248

[B27] Michail M. Davies J. E. Cameron J. D. Parker K. H. Brown A. J. (2018). Pathophysiological coronary and microcirculatory flow alterations in aortic stenosis. *Nat. Rev. Cardiol.* 15 420–431. 10.1038/s41569-018-0011-2 29713011

[B28] Ng J. Bourantas C. V. Torii R. Ang H. Y. Tenekecioglu E. Serruys P. W. (2017). Local hemodynamic forces after stenting: implications on restenosis and thrombosis. *Arterioscler. Thromb. Vasc. Biol.* 37 2231–2242. 10.1161/ATVBAHA.117.309728 29122816

[B29] O’Brien C. C. Lopes A. C. Kolandaivelu K. Kunio M. Brown J. Kolachalama V. B. (2016). Vascular response to experimental stent malapposition and under-expansion. *Ann. Biomed. Eng.* 44 2251–2260. 10.1007/s10439-015-1518-x 26732391 PMC4893976

[B30] Ostrowski M. A. Huang E. Y. Surya V. N. Poplawski C. Barakat J. M. Lin G. L. (2016). Multiplexed fluid flow device to study cellular response to tunable shear stress gradients. *Ann. Biomed. Eng.* 44 2261–2272. 10.1007/s10439-015-1500-7 26589597 PMC4874920

[B31] Ostrowski M. A. Huang N. F. Walker T. W. Verwijlen T. Poplawski C. Khoo A. S. (2014). Microvascular endothelial cells migrate upstream and align against the shear stress field created by impinging flow. *Biophys. J.* 106 366–374. 10.1016/j.bpj.2013.11.4502 24461011 PMC3907231

[B32] Palmaz J. C. Benson A. Sprague E. A. (1999). Influence of surface topography on endothelialization of intravascular metallic material. *J. Vasc. Interv. Radiol.* 10 439–444. 10.1016/S1051-0443(99)70063-1 10229473

[B33] Putra N. K. Wang Z. Anzai H. Ohta M. (2018). “Computational fluid dynamics analysis to predict endothelial cells migration during flow exposure experiment with placement of two stent wires,” in *Proceedings of the 2018 40th Annual International Conference of the IEEE Engineering in Medicine and Biology Society (EMBC)*, (Piscataway, NJ: IEEE), 5454–5457. 10.1109/EMBC.2018.8513517 30441571

[B34] Quilici J. Banzet N. Paule P. Meynard J. B. Mutin M. Bonnet J. L. (2004). Circulating endothelial cell count as a diagnostic marker for non-ST-elevation acute coronary syndromes. *Circulation* 110 1586–1591. 10.1161/01.CIR.0000142295.85740.98 15364807

[B35] Rikhtegar F. Wyss C. Stok K. S. Poulikakos D. Muller R. Kurtcuoglu V. (2014). Hemodynamics in coronary arteries with overlapping stents. *J. Biomech.* 47 505–511. 10.1016/j.jbiomech.2013.10.048 24275438

[B36] Souilhol C. Serbanovic-Canic J. Fragiadaki M. Chico T. J. Ridger V. Roddie H. (2020). Endothelial responses to shear stress in atherosclerosis: a novel role for developmental genes. *Nat. Rev. Cardiol.* 17 52–63. 10.1038/s41569-019-0239-5 31366922

[B37] Stamp M. E. Jotten A. M. Kudella P. W. Breyer D. Strobl F. G. Geislinger T. M. (2016). Exploring the limits of cell adhesion under shear stress within physiological conditions and beyond on a chip. *Diagnostics* 6:38. 10.3390/diagnostics6040038 27775638 PMC5192513

[B38] Tahir H. Bona-Casas C. Hoekstra A. G. (2013). Modelling the effect of a functional endothelium on the development of in-stent restenosis. *PLoS One* 8:e66138. 10.1371/journal.pone.0066138 23785479 PMC3681932

[B39] Tenekecioglu E. Torii R. Sotomi Y. Collet C. Dijkstra J. Miyazaki Y. (2017). The effect of strut protrusion on shear stress distribution: hemodynamic insights from a prospective clinical trial. *JACC Cardiovasc. Interv.* 10 1803–1805. 10.1016/j.jcin.2017.06.020 28882287

[B40] Tesfamariam B. (2016). Endothelial repair and regeneration following intimal injury. *J. Cardiovasc. Transl. Res.* 9 91–101. 10.1007/s12265-016-9677-1 26797874

[B41] Torii R. Tenekecioglu E. Katagiri Y. Chichareon P. Sotomi Y. Dijkstra J. (2020). The impact of plaque type on strut embedment/protrusion and shear stress distribution in bioresorbable scaffold. *Eur. Heart J. Cardiovasc. Imaging* 21 454–462. 10.1093/ehjci/jez155 31215995

[B42] Van der Heiden K. Gijsen F. J. Narracott A. Hsiao S. Halliday I. Gunn J. (2013). The effects of stenting on shear stress: relevance to endothelial injury and repair. *Cardiovasc. Res.* 99 269–275. 10.1093/cvr/cvt090 23592806

[B43] Wang J. Jin X. Huang Y. Ran X. Luo D. Yang D. (2018). Endovascular stent-induced alterations in host artery mechanical environments and their roles in stent restenosis and late thrombosis. *Regen. Biomater.* 5 177–187. 10.1093/rb/rby006 29942650 PMC6007795

[B44] Yoshino D. Sakamoto N. Sato M. J. I. B. (2017). Fluid shear stress combined with shear stress spatial gradients regulates vascular endothelial morphology. *Integr. Biol.* 9 584–594. 10.1039/c7ib00065k 28548171

[B45] Zhang M. Rehman J. Malik A. B. J. C. (2014). Endothelial progenitor cells and vascular repair. *Curr. Opin. Hematol.* 21 224–228. 10.1097/MOH.0000000000000041 24637956 PMC4090051

